# Inhibiting Biofilm Formation via Simultaneous Application of Nitric Oxide and Quorum Quenching Bacteria

**DOI:** 10.3390/membranes13100836

**Published:** 2023-10-20

**Authors:** Youkyoung Kim, Parthiban Anburajan, Hyeok Kim, Hyun-Suk Oh

**Affiliations:** 1 Department of Environmental Engineering, Seoul National University of Science and Technology, Seoul 01811, Republic of Korea; yoooukk@seoultech.ac.kr (Y.K.); anbubioenergy@seoultech.ac.kr (P.A.); idea1440@seoultech.ac.kr (H.K.); 2 Institute of Environmental Technology, Seoul National University of Science and Technology, Seoul 01811, Republic of Korea

**Keywords:** membrane filtration, nitric oxide, bacterial signaling, quorum quenching, biofilm, biofouling

## Abstract

Membrane biofouling is an inevitable challenge in membrane-based water treatment systems such as membrane bioreactors. Recent studies have shown that biological approaches based on bacterial signaling can effectively control biofilm formation. Quorum quenching (QQ) is known to inhibit biofilm growth by disrupting quorum sensing (QS) signaling, while nitric oxide (NO) signaling helps to disperse biofilms. In this study, batch biofilm experiments were conducted to investigate the impact of simultaneously applying NO signaling and QQ for biofilm control using *Pseudomonas aeruginosa* PAO1 as a model microorganism. The NO treatment involved the injection of NONOates (NO donor compounds) into mature biofilms, while QQ was implemented by immobilizing QQ bacteria (*Escherichia coli* TOP10-AiiO or *Rhodococcus* sp. BH4) in alginate or polyvinyl alcohol/alginate beads to preserve the QQ activity. When QQ beads were applied together with (Z)-1-[N-(3-aminopropyl)-N-(n-propyl) amino]diazen-1-ium-1,2-diolate (PAPA NONOate), they achieved a 39.0% to 71.3% reduction in biofilm formation, which was substantially higher compared to their individual applications (16.0% to 54.4%). These findings highlight the significant potential of combining QQ and NO technologies for effective biofilm control across a variety of processes that require enhanced biofilm inhibition.

## 1. Introduction

Membrane bioreactors (MBRs) combining biological treatment and membrane filtration processes allow for the efficient removal of suspended solids, organic matter, nutrients (such as nitrogen and phosphorus), and even micropollutants [1,2]. Moreover, MBRs have a smaller footprint and enable more efficient and stable operation compared to conventional wastewater treatments. However, one of the main issues affecting the operation of MBRs is membrane biofouling, which is associated with biofilm formation [3,4]. Biofilm formation on the membrane surface can lead to fouling, which reduces the permeability of the membrane and thus the efficiency of the system. Fouling can result in increased transmembrane pressure, which requires more frequent cleaning and maintenance, resulting in higher operational costs [5,6]. Extensive research has been conducted over the past three decades to address the challenges posed by biofouling through material, physical, chemical, and operational approaches. These techniques encompass the development of novel membrane materials; alterations to the membrane surface and structure; the control of bubble size in aeration; backwashing; the addition of adsorbents; and adjustments to operational parameters such as mixed liquor suspended solids, solid retention time, critical flux, and more [7,8]. However, because it is nearly impossible to completely prevent biofilm formation, it is important to develop a technology that can effectively minimize and control this process.

Having recognized this limitation, a recently emerged research direction focuses on the modulation of cell–cell signaling between microorganisms. Recent studies have highlighted the contribution of nitric oxide (NO) in regulating the expression of the specific genes and signaling pathways related to biofilm dispersal [9,10]. Understanding the mechanisms underlying NO signaling in biofilm dispersions holds great promise for developing novel strategies to control and mitigate biofilm-related issues in various applications, including water treatment, medical devices, cooling towers, etc. Bacteria sense NO through a signal response pathway, which stimulates intracellular phosphodiesterase activity. This activity leads to the degradation of cyclic diguanylate monophosphate (c-di-GMP) and induces changes in gene expression that favor the planktonic state [11,12]. Thus, NO plays a crucial role in the transition of microorganisms from attached to suspended growth. Owing to its unstable gas nature with a very short half-life of 2–6 s in the radical state, injecting an NO donor that gradually releases NO into water at a neutral pH can achieve efficient biofilm dispersion [8,13,14]. Several studies have demonstrated the biofilm inhibition effect of NONOates, which have the chemical formula R^1^R^2^N−(NO^−^)−N=O, where R^1^ and R^2^ represent alkyl groups, in both batch biofilm test and continuous water treatment systems [9,15,16,17].

Quorum sensing (QS) is another important bacterial signaling mechanism in biofilm formation. QS is a cell-to-cell communication mechanism used by bacteria to regulate gene expression based on their density, thus coordinating various group behaviors, including virulence factor secretion, motility, and biofilm formation. This includes the production and sensing of signaling molecules known as autoinducers, such as N-acyl homoserine lactones (AHLs), autoinducer-2, and autoinducing peptides [18]. Disrupting bacterial QS, known as quorum quenching (QQ), can be an effective method to inhibit biofilm formation. In particular, extensive research on controlling biofouling in membrane processes using QQ bacteria that disrupt the QS process of AHLs has been carried out in the past decade [19,20]. Specifically, the technique of entrapping QQ bacteria within hydrogel beads has gained popularity as a means of implementing the QQ strategy in continuous water treatment processes [15,21]. This method safeguards the bacteria from environmental stress and competition with other microorganisms.

Biofilm formation involves a series of processes, including initial attachment, growth, and dispersal [22]. While QS is known to influence the initial attachment and growth, NO has been reported to play a role in biofilm dispersal. However, the simultaneous use of QQ and NO-based approaches in biofilm control has not been explored. While both of these technologies are effective in biofilm control, it is important to investigate whether combining them can indeed ensure more effective control, given that they participate in different stages of biofilm formation. Therefore, this study aims to investigate the combined effect of QQ and NO signaling mechanisms on biofilm control. In batch tests using *Pseudomonas aeruginosa* as a model biofilm-forming bacterium, we applied combinations of two QQ bacteria (*Rhodococcus* sp. BH4 and genetically modified *Escherichia coli*) with various types of NONOates to investigate their efficiency in controlling biofilms. The results of this study illustrate the potential and applicability of combining the two signaling-based approaches in overcoming the challenge of inhibiting biofilm formation.

## 2. Materials and Methods

### 2.1. Microorganisms

The strains, plasmids, and culture conditions used in our experiments are listed in Table 1. All strains were cultured in Luria–Bertani (LB) broth (BD Difco, Detroit, MI, USA). To investigate the regulation of biofilm formation via QS, we cultured *P. aeruginosa* PAO1, which is an aerobic Gram-negative bacterium known to produce biofilms through the QS mechanism. The suppression of the QS of AHL-signaling molecules was confirmed using *Rhodococcus* sp. BH4 and *E. coli* TOP10-AiiO. *E. coli* TOP10-Empty was employed as a negative control for *E. coli* TOP10-AiiO. Furthermore, *Agrobacterium tumefaciens* A136 (Ti^−^)(pCF218)(pCF372) was utilized as a biosensor to detect the presence of *N*-(3-oxo-hexanoyl)-L-homoserine lactone (OHHL).

### 2.2. NO Donors for Biofilm Dispersal

Various NONOates, which can release NO spontaneously at ambient temperatures, were used as NO donors. (Z)-1-[*N*-(3-aminopropyl)-*N*-(*n*-propyl)amino]diazen-1-ium-1,2-diolate (PAPA NONOate, half-life: 15 min), (Z)-1-[*N*-[3-aminopropyl]-*N*-[4-(3-aminopropylammonio)butyl]-amino]diazen-1-ium-1,2-diolate (Spermine NONOate, half-life: 39 min), and (Z)-1-[*N*-(3-aminopropyl)-*N*-(3-ammoniopropyl)amino]diazen-1-ium-1,2-diolate (DPTA NONOate, half-life: 3 h) were purchased from Cayman Chemical (Ann Arbor, MI, USA). Stock solutions of the NO donors were prepared by dissolving them in 10 mM NaOH to prevent NO release before the experiment.

### 2.3. Preparation of QQ Beads

**Figure 1:**
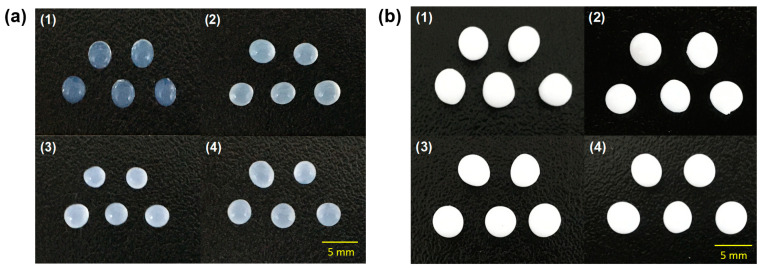
Photographs of (**a**) alginate and (**b**) PVA/alginate beads. (1) vacant beads. (2) *Rhodococcus* sp. BH4 beads. (3) *E. coli* TOP10-Empty beads. (4) *E. coli* TOP10-AiiO beads).

Two kinds of hydrogel beads, alginate and polyvinyl alcohol (PVA)/alginate, were used to immobilize QQ bacteria (*E. coli* TOP10-AiiO or *Rhodococcus* sp. BH4) at an OD_600_ of 3. The alginate beads were prepared based on a previously reported method in which a 1% (*w*/*v*) alginate–bacterial cell mixture was dropped into a 4% (*w*/*v*) calcium chloride solution [25]. A modified version of a previous method was used for preparing the PVA/alginate beads: a 10% (*w*/*v*) PVA (Sigma-Aldrich, St Louis, MO, USA) and 1% (*w*/*v*) alginate–bacterial cell mixture was dropped onto a 7% (*w*/*v*) boric acid and 4% (*w*/*v*) calcium chloride solution, followed by incubation for 1 h for the first cross-linking process [27]. After that, the beads were transferred to a 0.5 M sodium sulfate solution and incubated for 8 h for the second cross-linking process. Figure 1 shows photographs of the prepared beads.

### 2.4. Biofilm Formation Assay

The impact of NO and/or QQ on the biofilm formation of *P. aeruginosa* PAO1 was assessed through 24-well microtiter plate assays, which were conducted following previously reported procedures [20]. The model biofilm-forming bacterium *P. aeruginosa* PAO1 was grown in LB broth overnight and centrifuged at 4500 rpm for 10 min. Then, the concentration of *P. aeruginosa* PAO1 was adjusted to 0.03 of OD_600_ using M9 minimal medium (which contained 9 mM NaCl_2_, 22 mM KH_2_PO_4_, 48 mM Na_2_HPO_4_, 19 mM NH_4_Cl, 2 mM MgSO_4_, 100 μM CaCl_2_, and 0.4% glucose, pH 7.0). The bacterial solutions (1 mL) were placed in the wells of a 24-well microtiter plate and incubated for 6 h at 30 °C with shaking (180 rpm). After 6 h of culture, 10 mM NaOH (Control) or the prepared NO donor stock solution was added to each well to obtain final concentrations of 50 and 100 μM. The plate was incubated with shaking for an additional 30 min after the injection of the NO donor.

**Figure 2:**
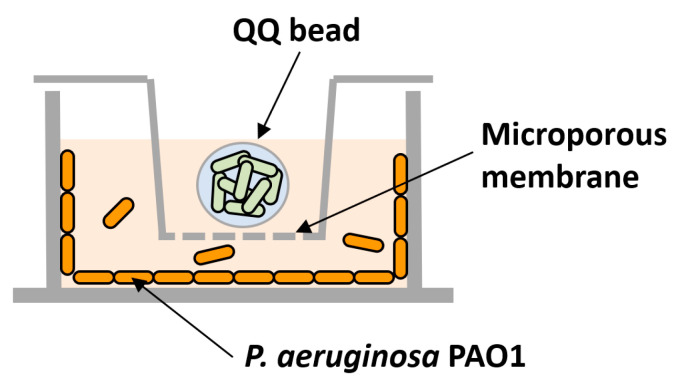
Schematic diagram of biofilm test using a microwell plate and a transwell system. The model biofilm-forming organism, *P. aeruginosa* PAO1, was inoculated into the space below the transwell, and quorum quenching (QQ) beads were placed inside the transwell.

To test the individual or combined effects of QQ, transwells (6.5 mm with 8.0 μm polycarbonate membrane inserts, Coster) were used to prevent the QQ beads from directly contacting the 24-well plates (Figure 2). Following the inoculation of *P. aeruginosa* PAO1 as described above, transwells were installed in the 24-well plate; then, a QQ and a control bead (immobilizing *E. coli* TOP10-Empty) were placed into the corresponding transwell.

The biofilms formed on the well surface were quantified using crystal violet (CV) assays. The biofilm remaining in the wells was stained using 0.1% (*w*/*v*) CV for 20 min and washed two times with phosphate-buffered saline buffer. The wells were then destained with 99.9% ethanol, and the quantity of biofilm was determined by measuring the absorbance at a wavelength of 550 nm using a microtiter plate reader (Gen 5, Biotek, Winooski, VT, USA).

The expected combined effect of biofilm reduction was estimated using the following [28]:

R_exp_ = 1 − (1 − R_QQ_) × (1 − R_NO_) = R_QQ_ + R_NO_ − R_QQ_ × R_NO_ (1)

where R_exp_, R_QQ_, and R_NO_ denote the expected biofilm reduction (%) by the combination of QQ and NO, the biofilm reduction (%) by QQ, and the biofilm reduction (%) by NO, respectively.

### 2.5. Bioassay for the Quantification of AHLs

AHLs were quantified using a luminescence-based bioassay method that has been described in a previous study [20]. The samples (5 μL) and the reporter strain A136 (95 μL) were mixed into a 96-well plate and then incubated at 30 °C for 90 min. Then, 30 μL of Beta-Glo assay system (Promega, Madison, WI, USA) was added, and the plate was kept at 25 °C for 35 min. The luminescence intensity was measured using a microplate reader (Synergy HTX, Biotek, Winooski, VT, USA). OHHL was dissolved in a 50 mM Tris-HCl buffer to prepare standard solutions at concentrations of 12.5, 25, 50, 100, and 200 nM.

### 2.6. QQ Activity Test

QQ activity tests were conducted to confirm the AHL degradation capability of the QQ beads. Twenty milliliters of 200 nM OHHL were placed into a conical tube, along with 50 QQ beads. The mixture was cultured at 30 °C and 100 rpm, and 500-μL samples were collected at reaction times of 0, 1, 10, 30, 60, 120, and 180 min. These samples were centrifuged at 13,500 rpm for 3 min, and 200 μL of the supernatant was treated at 95 °C for 2 min before storing it at −20 °C. Then, the residual OHHL concentrations at each time point were measured using the AHL bioassay method described in Section 2.5.

The rate coefficient of QQ was expressed as a pseudo-first-order reaction according to the degradation rate of the OHHL at time *t* [16]:

(2) $$\frac{d[AHL]}{dt}=k[AHL]$$

(3) $$ln\frac{{\left[AHL\right]}_{t}}{{\left[AHL\right]}_{0}}=kt$$

## 3. Results and Discussion

### 3.1. Effect of QQ Beads on Biofilm Formation

**Figure 3:**
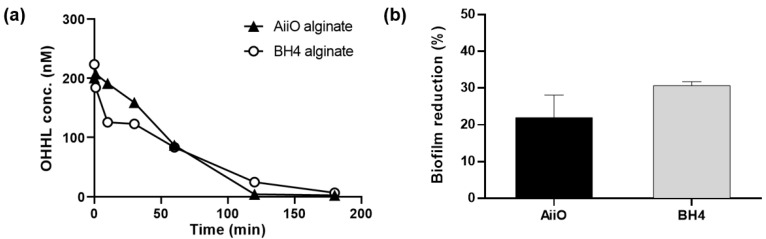
Effect of quorum quenching alginate bead. (**a**) OHHL degradation rates of alginate beads entrapping *E. coli* TOP10-AiiO or *Rhodococcus* sp. BH4. (**b**) Biofilm reduction efficiency of AiiO and BH4 alginate beads. Error bars represent standard deviations (*n* = 2).

The QQ activity of alginate beads entrapping *E. coli* TOP10-AiiO or *Rhodococcus* sp. BH4 strains was measured as shown in Figure 3a. In the case of the AiiO alginate beads, almost all OHHL was decomposed in 120 min, while complete decomposition with the BH4 alginate beads required 180 min. These were substantially slower rates compared to when *E. coli* TOP10-AiiO or *Rhodococcus* sp. BH4 was tested in suspension, both of which completely degraded OHHL within several minutes [19,25]. This is because the rate at which AHL diffused into the beads’ interior affected the overall degradation rate. The rate coefficient for the degradation of OHHL was higher in AiiO alginate beads (1.98 h^−1^) compared to BH4 alginate beads (0.95 h^−1^), resulting in the complete degradation of 200 nM OHHL in 120 and 180 min, respectively. While there was a difference in the AHL degradation rate between the two beads, both beads effectively degraded AHL. Although the rate of AHL degradation may decrease when microbial cells are confined within hydrogel beads compared to when they are in a suspended state, the protection of QQ bacteria can provide a safer environment for continuous process applications [29].

To evaluate the efficacy of biofilm reduction by the QQ beads, a biofilm test was conducted using a microwell plate setup with a transwell, as shown in Figure 2. As shown in Figure 3b, the addition of an AiiO alginate bead led to a 21.9% reduction in biofilm formation compared to the addition of an Empty alginate bead (i.e., the alginate bead entrapping *E. coli* TOP10-Empty). The addition of the BH4 alginate bead also yielded a substantial biofilm reduction (30.7%) compared to the vacant bead (i.e., the alginate bead without bacteria). In any case, the results confirmed that quenching AHL using alginate beads with immobilized *E. coli* TOP10-AiiO or *Rhodococcus* sp. BH4 can mitigate the biofilm formation of *P. aeruginosa* PAO1.

### 3.2. Dispersal of Biofilm by Addition of NO Donors

To assess the NO-induced dispersal of biofilm cells, we selected three specific NO donors: PAPA NONOate, Spermine NONOate, and DPTA NONOate. These compounds have half-lives in water ranging from several minutes to a few hours, which are neither too short nor too long [9]. Thus, they are considered to strike an appropriate balance in terms of the rate and long-term stability of NO emissions.

**Figure 4:**
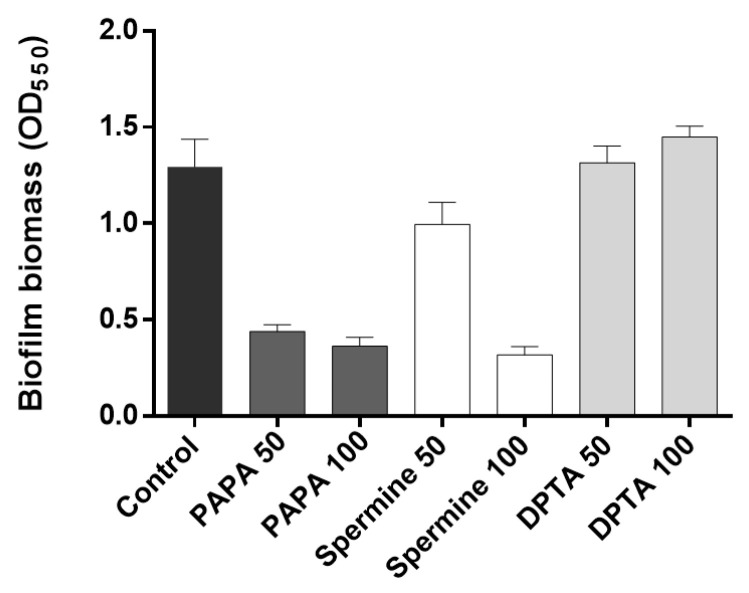
Biofilm inhibition by nitric oxide (NO) donors. PAPA 50 and PAPA 100 indicate the addition of 50 μM and 100 μM PAPA NONOate, respectively. The same labeling approach was followed for other substances (Spermine NONOate and DPTA NONOate) as well. Error bars represent standard deviations (*n* = 3).

The effectiveness of different types and concentrations of NO donors in mitigating biofilm formation was assessed by quantifying the percentage of biofilm reduction following a 30 min incubation period with NO donors. The results showed that both 50 μM and 100 μM PAPA NONOate additions led to substantial reductions (65.9% and 71.8%, respectively) in biofilm formation (Figure 4). This was a greater reduction than when an AiiO alginate bead (21.9%) or a BH4 alginate bead (30.7%) was injected, as shown in Figure 3b. In the case of Spermine NONOate, a 50 μM injection resulted in 23% biofilm reduction, while a 100 μM injection achieved a significantly improved biofilm reduction of 75.3%. In contrast, DPTA NONOate showed no reduction in biofilm formation at either concentration during the 30 min incubation period, consistent with previous findings [9]. Overall, PAPA NONOate demonstrated superior biofilm reduction effects at 50–100 μM injections compared to the other two NO donors; thus, it was chosen for the subsequent experiments.

### 3.3. Combination of NO Treatment and QQ Alginate Beads for Biofilm Reduction

**Figure 5:**
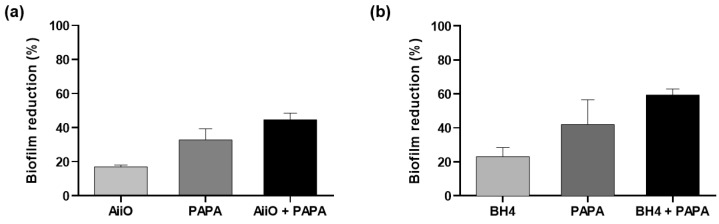
The combination effect of QQ alginate beads and NO donor on the biofilm reduction. (**a**) Biofilm reduction by adding an AiiO alginate bead, 50 μM PAPA NONOate, and their combination. (**b**) Biofilm reduction by adding a BH4 alginate bead, 50 μM PAPA NONOate, and their combination. Error bars represent standard deviations (*n* = 3).

After confirming the biofilm reduction effects of the individual QQ and NO treatments, we investigated their combined inhibitory effect on biofilm formation. As shown in Figure 5a, when an AiiO alginate bead and 50 μM PAPA NONOate were applied separately, the biofilm removal rates were approximately 16.9% and 32.8%, respectively. The expected reduction efficiency of the combination of these two treatments was estimated to be 44.2% using Equation (1). An experiment with an AiiO alginate bead applied together with 50 μM PAPA NONOate showed a 44.6% reduction in biofilm formation (Figure 5a). This value was close to the theoretical prediction, indicating that no synergistic or antagonistic effect was observed using the combination of the two treatments.

The BH4 alginate beads also exhibited a similar trend when combined with AiiO alginate beads and PAPA NONOate beads. When a BH4 alginate bead and 50 μM PAPA NONOate were applied separately, they showed 23.1% and 42.0% reductions in biofilm formation, respectively (Figure 5b). When they were combined, a 59.4% reduction in biofilm formation was achieved, which is slightly lower than the expected rate of 64.2%. Therefore, we can also conclude that no synergistic effect was present between the BH4 alginate bead and 50 μM PAPA NONOate.

**Figure 6:**
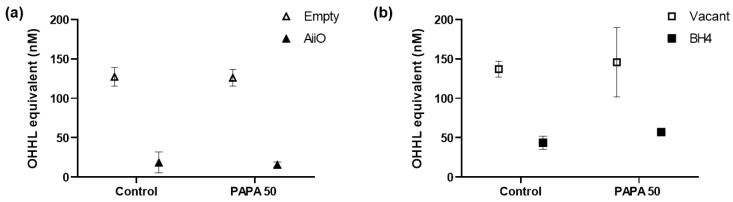
OHHL concentration in supernatant after 6 h of biofilm test. (**a**) Combination of an AiiO alginate bead and PAPA NONOate. NaOH (Control) or 50 μM PAPA NONOate were added together with an alginate bead entrapping *E. coli* TOP10-Empty or *E. coli* TOP10-AiiO. (**b**) Combination of a BH4 alginate bead and PAPA NONOate. NaOH (Control) or 50 μM PAPA NONOate were added together with a vacant alginate bead or an alginate bead entrapping *Rhodococcus* sp. BH4. Error bars represent standard deviations (*n* = 3).

Figure 6 shows the amount of AHL QS signaling molecules that were degraded by QQ in the biofilm reduction experiments. *P. aeruginosa* PAO1 is known to produce more than two types of AHLs, including *N*-butanoyl-homoserine lactone and *N*-3-oxo-dodecanoyl-homoserine lactone. The biosensor A136 used in this experiment can respond to various types of AHLs; therefore, bioassays using this biosensor cannot determine which AHLs were generated and degraded. However, the luminescence values obtained from the sample can be represented as OHHL equivalent values using the standard curve established with OHHL [25].

In the absence of a PAPA NONOate injection (control), an 85.3% reduction in AHL levels was observed upon the injection of an AiiO alginate bead compared to the well with an Empty alginate bead (Figure 6a). This confirms that the AHLs produced by *P. aeruginosa* PAO1 within the transwell microplate setup were effectively degraded by the AiiO bead. The injection of PAPA NONOate did not have any impact on either the production or degradation of the AHLs. In the case of the BH4 alginate bead, 68.2% of the AHLs were found to be degraded compared to the well where a vacant bead was injected (Figure 6b, control). Although this degradation rate was somewhat lower than that observed with the AiiO bead, it is expected to have a significant impact on reducing biofilm formation by degrading a substantial amount of the AHLs. The injection of PAPA NONOate also did not affect the AHL production and degradation in the experiment with the BH4 bead. Despite the high AHL removal rate (68.2–85.3%), the efficiency of biofilm reduction by the QQ beads (16.9–23.1%) was lower than expected.

This discrepancy could be attributed to *P. aeruginosa* PAO1 utilizing an extracellular signal molecule called *Pseudomonas* quinolone signal (PQS) in addition to AHL. PQS influences the synthesis of QS-dependent extracellular products such as pyocyanin and elastase, which are associated with biofilm formation [30]. Moreover, while AHL was effectively removed from the supernatant, the possibility that AHL remained in high concentrations within the extracellular polymeric substance (EPS) matrix of the biofilm cannot be ruled out [31].

In summary, the QQ beads effectively degraded the AHLs produced by *P. aeruginosa* PAO1, thus inhibiting biofilm formation, although the reduction rate was limited (16.9–23.1%). However, when NO was applied together with QQ beads, the reduction rate increased to 44.6–59%, indicating that the combination of the two techniques can achieve more effective biofilm reduction.

### 3.4. Combination of NO Treatment and QQ PVA/Alginate Beads

Despite their high biocompatibility and ease of preparation, alginate beads have the disadvantages of low physical strength and stability [32,33,34]. Therefore, to improve the durability of the QQ beads, we produced beads by immobilizing two QQ bacteria using a mixture of PVA and alginate. The PVA/alginate beads, like the alginate beads, also had a spherical shape, and there were no significant differences in their shape (Figure 1). However, the vacant beads made with PVA/alginate appeared opaque and white, in contrast to the translucent nature of the vacant alginate beads. Furthermore, the PVA/alginate beads were fabricated to be larger than the alginate beads. This was due to an increase in polymer viscosity caused by mixing PVA with alginate. Polymer viscosity is greatly influenced by temperature, allowing for bead size control by adjusting the polymer temperature. However, in this study, both types of beads were produced under the same temperature conditions (i.e., room temperature) as the aim of this study was not to quantitatively compare the two types of beads.

**Figure 7:**
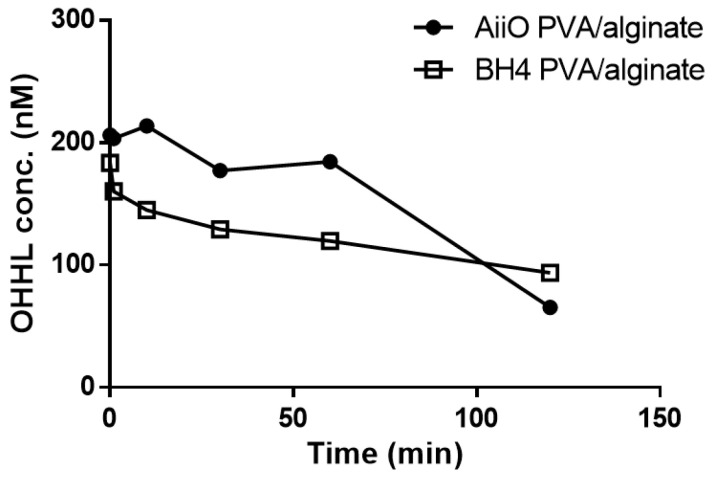
Degradation of OHHL by PVA/alginate beads entrapping *E. coli* TOP10-AiiO or *Rhodococcus* sp. BH4.

As expected, the addition of PVA resulted in beads with higher physical strength and stability, but their AHL degradation ability showed a slight decrease (Figure 7). Over 120 min, BH4 and AiiO PVA/alginate beads degraded approximately 48.5% and 65.7% of OHHL, respectively. The rate coefficients for the degradation of OHHL by the AiiO PVA/alginate beads and BH4 PVA/alginate beads were 0.57 and 0.25 h^−1^, respectively. Compared to the alginate beads, these are somewhat reduced values, and the key trend observed is an unavoidable trade-off that occurs as the physical strength of the bead increases.

**Figure 8:**
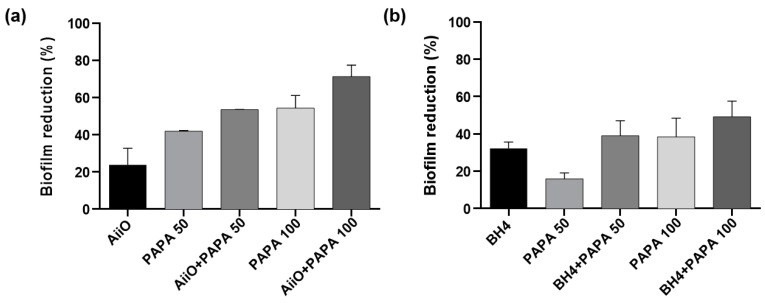
The combined effect of QQ PVA/alginate beads and NO donor on biofilm reduction. (**a**) Biofilm reduction after adding a AiiO PVA/alginate bead, PAPA NONOate (50 or 100 μM), and their combination. (**b**) Biofilm reduction after adding a BH4 PVA/alginate bead, PAPA NONOate (50 or 100 μM), and their combination. Error bars represent standard deviations (*n* = 3).

As shown in Figure 8, a similar trend to those observed using the alginate beads was obtained when the PVA/alginate beads were combined with PAPA NONOate. The combination of an AiiO PVA/alginate bead and 50 μM PAPA NONOate resulted in a 53.5% reduction in biofilm formation (Figure 8a), which was nearly identical to the expected reduction of 55.9% calculated based on the combination using Equation (1). Similarly, a 39.0% reduction in biofilm formation was observed when a BH4 PVA/alginate bead and 50 μM PAPA NONOate were applied together (Figure 8b), which was comparable to the expected reduction of 43.1%. To achieve even higher biofilm reduction rates, the dosage of PAPA NONOate was increased to 100 μM. Its combination with an AiiO PVA/alginate bead led to a biofilm reduction of up to 71.3%, whereas that with BH4 yielded a reduction of up to 49.2%. These results confirmed that through using durable PVA/alginate beads with potential applications in MBR processes for wastewater treatment, the combination of immobilized QQ bacteria with an NO donor can further enhance the biofilm reduction effect.

## 4. Conclusions

This study investigated the combination of QQ and NO signaling for effective biofilm control. Biofilm tests were performed using a microplate equipped with a transwell that confirmed that biofilm reduction occurred when the QQ bacteria-immobilized beads and NO donors were applied separately. When these two approaches were combined, they led to a more significant biofilm reduction compared to the outcomes of their individual applications, thus demonstrating the effectiveness of their combination. By comparing the results with the expected combined effect calculated mathematically, no synergistic or antagonistic effects were observed in their combination. Although this research study has limitations in terms of its use of a monospecies microorganism for a batch-type biofilm test, the results highlight the promising prospects of combining these two technologies for more efficient biofilm inhibition in various processes requiring biofilm control, including membrane filtration processes. By exploring these possibilities and confirming their effectiveness in future laboratory- or pilot-scale water treatment processes, we can assess their practical applicability in full-scale processes.

## Figures and Tables

**Table 1:** Bacteria used for this study.

Strain	Purpose and Characteristics	Incubation Temp. (°C)	Antibiotics	Source
*P. aeruginosa* PAO1	Model biofilm strain. Wild type	37	-	[23]
*Rhodococcus* sp. BH4	AHL quencher strain. Wild type	30	-	[24]
*E. coli* TOP10-Empty	Control strain carrying pTrcHis2 plasmid; Kan^R^, Amp^R^	37	Kanamycin (50 μg/mL)	[25]
*E. coli* TOP10-AiiO	AHL quencher strain carrying pTriHis2-AiiO plasmid; Kan^R^, Amp^R^	37	Kanamycin (50 μg/mL)	[25]
*A. tumefaciens* A136 (Ti^−^)(pCF218)(pCF372)	AHL biosensor, carrying pCF218 plasmid with *traR* and pCF372 plasmid with *PtraI-lacZ*; Sp^R^, Tet^R^	30	Spectinomycin (50 μg/mL), Tetracyclin (4.5 μg/mL)	[26]

## Data Availability

The data that support the findings of this study are available on request from the corresponding author. The data are not publicly available due to privacy or ethical restrictions.

## References

[1] Drews A. (2010). Membrane Fouling in Membrane Bioreactors-Characterisation, Contradictions, Cause and Cures. J. Membr. Sci..

[2] Smith A.L., Stadler L.B., Love N.G., Skerlos S.J., Raskin L. (2012). Perspectives on Anaerobic Membrane Bioreactor Treatment of Domestic Wastewater: A Critical Review. Bioresour. Technol..

[3] Shahid M.K., Kashif A., Rout P.R., Aslam M., Fuwad A., Choi Y., Banu J.R., Park J.H., Kumar G. (2020). A Brief Review of Anaerobic Membrane Bioreactors Emphasizing Recent Advancements, Fouling Issues and Future Perspectives. J. Environ. Manag..

[4] Lee W.N., Chang I.S., Hwang B.K., Park P.K., Lee C.H., Huang X. (2007). Changes in Biofilm Architecture with Addition of Membrane Fouling Reducer in a Membrane Bioreactor. Process Biochem..

[5] Jegatheesan V., Pramanik B.K., Chen J., Navaratna D., Chang C.Y., Shu L. (2016). Treatment of Textile Wastewater with Membrane Bioreactor: A Critical Review. Bioresour. Technol..

[6] Krzeminski P., Leverette L., Malamis S., Katsou E. (2017). Membrane Bioreactors—A Review on Recent Developments in Energy Reduction, Fouling Control, Novel Configurations, LCA and Market Prospects. J. Membr. Sci..

[7] Le-Clech P., Chen V., Fane T.A.G. (2006). Fouling in Membrane Bioreactors Used in Wastewater Treatment. J. Membr. Sci..

[8] Xiong Y., Liu Y. (2010). Biological Control of Microbial Attachment: A Promising Alternative for Mitigating Membrane Biofouling. Appl. Microbiol. Biotechnol..

[9] Oh H.S., Constancias F., Ramasamy C., Tang P.Y.P., Yee M.O., Fane A.G., McDougald D., Rice S.A. (2018). Biofouling Control in Reverse Osmosis by Nitric Oxide Treatment and Its Impact on the Bacterial Community. J. Membr. Sci..

[10] McDougald D., Rice S.A., Barraud N., Steinberg P.D., Kjelleberg S. (2012). Should We Stay or Should We Go: Mechanisms and Ecological Consequences for Biofilm Dispersal. Nat. Rev. Microbiol..

[11] Cai Y.M., Webb J.S. (2020). Optimization of Nitric Oxide Donors for Investigating Biofilm Dispersal Response in *Pseudomonas aeruginosa* Clinical Isolates. Appl. Microbiol. Biotechnol..

[12] Barraud N., Storey M.V., Moore Z.P., Webb J.S., Rice S.A., Kjelleberg S. (2009). Nitric Oxide-Mediated Dispersal in Single- and Multi-Species Biofilms of Clinically and Industrially Relevant Microorganisms. Microb. Biotechnol..

[13] Heil J., Vereecken H., Brüggemann N. (2016). A Review of Chemical Reactions of Nitrification Intermediates and Their Role in Nitrogen Cycling and Nitrogen Trace Gas Formation in Soil. Eur. J. Soil Sci..

[14] Barraud N., Schleheck D., Klebensberger J., Webb J.S., Hassett D.J., Rice S.A., Kjelleberg S. (2009). Nitric Oxide Signaling in *Pseudomonas aeruginosa* Biofilms Mediates Phosphodiesterase Activity, Decreased Cyclic Di-GMP Levels, and Enhanced Dispersal. J. Bacteriol..

[15] Luo J., Zhang J., Barnes R.J., Tan X., Mcdougald D., Fane A.G., Zhuang G., Kjelleberg S., Cohen Y., Rice S.A. (2015). The Application of Nitric Oxide to Control Biofouling of Membrane Bioreactors. Microb. Biotechnol..

[16] Barnes R.J., Bandi R.R., Wong W.S., Barraud N., McDougald D., Fane A., Kjelleberg S., Rice S.A. (2013). Optimal Dosing Regimen of Nitric Oxide Donor Compounds for the Reduction of *Pseudomonas aeruginosa* Biofilm and Isolates from Wastewater Membranes. Biofouling.

[17] Barnes R.J., Bandi R.R., Chua F., Low J.H., Aung T., Barraud N., Fane A.G., Kjelleberg S., Rice S.A. (2014). The Roles of *Pseudomonas aeruginosa* Extracellular Polysaccharides in Biofouling of Reverse Osmosis Membranes and Nitric Oxide Induced Dispersal. J. Membr. Sci..

[18] Wang Y., Bian Z., Wang Y. (2022). Biofilm Formation and Inhibition Mediated by Bacterial Quorum Sensing. Appl. Microbiol. Biotechnol..

[19] Anburajan P., Kim Y., Rice S.A., Oh H.S. (2021). Bacterial Signaling and Signal Responses as Key Factors in Water and Wastewater Treatment. J. Water Process Eng..

[20] Noori A., Kim H., Kim M.H., Kim K., Lee K., Oh H.S. (2022). Quorum Quenching Bacteria Isolated from Industrial Wastewater Sludge to Control Membrane Biofouling. Bioresour. Technol..

[21] Lee K., Yu H., Zhang X., Choo K.H. (2018). Quorum Sensing and Quenching in Membrane Bioreactors: Opportunities and Challenges for Biofouling Control. Bioresour. Technol..

[22] Passos da Silva D., Schofield M.C., Parsek M.R., Tseng B.S. (2017). An Update on the Sociomicrobiology of Quorum Sensing in Gram-Negative Biofilm Development. Pathogens.

[23] Evans L.R., Linker A. (1973). Production and Characterization of the Slime Polysaccharide of *Pseudomonas aeruginosa*. J. Bacteriol..

[24] Ryu D.H., Lee S.W., Mikolaityte V., Kim Y.W., Jeong H., Lee S.J., Lee C.H., Lee J.K. (2020). Identification of a Second Type of Ahllactonase from Rhodococcus Sp. BH4, Belonging to the α/β Hydrolase Superfamily. J. Microbiol. Biotechnol..

[25] Oh H.S., Tan C.H., Low J.H., Rzechowicz M., Siddiqui M.F., Winters H., Kjelleberg S., Fane A.G., Rice S.A. (2017). Quorum Quenching Bacteria Can Be Used to Inhibit the Biofouling of Reverse Osmosis Membranes. Water Res..

[26] Fuqua C., Winans S.C. (1996). Conserved Cis-Acting Promoter Elements Are Required for Density-Dependent Transcription of Agrobacterium Tumefaciens Conjugal Transfer Genes. J. Bacteriol..

[27] Lee K., Kim Y.W., Lee S., Lee S.H., Nahm C.H., Kwon H., Park P.K., Choo K.H., Koyuncu I., Drews A. (2018). Stopping Autoinducer-2 Chatter by Means of an Indigenous Bacterium (*Acinetobacter* Sp. DKY-1): A New Antibiofouling Strategy in a Membrane Bioreactor for Wastewater Treatment. Environ. Sci. Technol..

[28] Duarte D., Vale N. (2022). Evaluation of Synergism in Drug Combinations and Reference Models for Future Orientations in Oncology. Curr. Res. Pharmacol. Drug Discov..

[29] Syafiuddin A., Boopathy R., Mehmood M.A. (2021). Recent Advances on Bacterial Quorum Quenching as an Effective Strategy to Control Biofouling in Membrane Bioreactors. Bioresour. Technol. Rep..

[30] Diggle S.P., Winzer K., Chhabra S.R., Worrall K.E., Cámara M., Williams P. (2003). The *Pseudomonas aeruginosa* Quinolone Signal Molecule Overcomes the Cell Density-Dependency of the Quorum Sensing Hierarchy, Regulates Rhl-Dependent Genes at the Onset of Stationary Phase and Can Be Produced in the Absence of LasR. Mol. Microbiol..

[31] Tan C.H., Oh H.S., Sheraton V.M., Mancini E., Joachim Loo S.C., Kjelleberg S., Sloot P.M.A., Rice S.A. (2020). Convection and the Extracellular Matrix Dictate Inter- And Intra-Biofilm Quorum Sensing Communication in Environmental Systems. Environ. Sci. Technol..

[32] Zain N.A.M., Suhaimi M.S., Idris A. (2011). Development and Modification of PVA-Alginate as a Suitable Immobilization Matrix. Process Biochem..

[33] Elwakeel K.Z., Ahmed M.M., Akhdhar A., Sulaiman M.G.M., Khan Z.A. (2022). Recent Advances in Alginate-Based Adsorbents for Heavy Metal Retention from Water: A Review. Desalin. Water Treat..

[34] Bai Y., Wu W. (2022). The Neutral Protease Immobilization: Physical Characterization of Sodium Alginate-Chitosan Gel Beads. Appl. Biochem. Biotechnol..

